# Static analysis of rectangular nanoplates using trigonometric shear deformation theory based on nonlocal elasticity theory

**DOI:** 10.3762/bjnano.4.109

**Published:** 2013-12-30

**Authors:** Mohammad Rahim Nami, Maziar Janghorban

**Affiliations:** 1 School of Mechanical Engineering, Shiraz University, Shiraz, Iran

**Keywords:** nonlocal elasticity theory, rectangular nanoplate, static analysis, trigonometric shear deformation theory

## Abstract

In this article, a new higher order shear deformation theory based on trigonometric shear deformation theory is developed. In order to consider the size effects, the nonlocal elasticity theory is used. An analytical method is adopted to solve the governing equations for static analysis of simply supported nanoplates. In the present theory, the transverse shear stresses satisfy the traction free boundary conditions of the rectangular plates and these stresses can be calculated from the constitutive equations. The effects of different parameters such as nonlocal parameter and aspect ratio are investigated on both nondimensional deflections and deflection ratios. It may be important to mention that the present formulations are general and can be used for isotropic, orthotropic and anisotropic nanoplates.

## Introduction

In recent years, some new higher-order shear deformation theories have been adopted for studying macro structures such as plates and shells [1]. Ghugal and Sayyad [2] proposed a trigonometric shear deformation theory (TSDT) to consider the influences of transverse deformation and normal strain. The displacements in the x- and y-directions were assumed to have a sinusoidal form in terms of thickness coordinate of the plate in order to consider the effects of shear deformation. Moreover, for the displacement in the z-direction, cosine form was used to see the influences of transverse normal strain. Ghugal and Sayyad [3] also calculated the frequencies of orthotropic rectangular plates on the basis of trigonometric shear deformation theory. The above mentioned methodology could be used for both thick and thin rectangular plates similar to 3D elasticity theory. In this theory, the transverse shear stresses satisfied the traction free boundary conditions of the rectangular plates and these stresses could be calculated from the constitutive equations. Ameur et al. [4] presented a trigonometric shear deformation theory with considering several unknown functions for the displacement field to study the static analysis of functionally graded plates. In this study, the effects of Pasternak and Winkler foundations were also investigated for functionally graded plates. Similar to the most of studies on FG structures, the material properties were assumed to vary in the thickness direction according to the power law distribution. Mantari et al. [5] modeled a new type of trigonometric shear deformation theory (TSDT) for studying plates with different material properties such as sandwich plates. It is assumed that this new type of displacement field depends on a parameter which is calculated according to the results of three dimensional elasticity theory. Kharde et al. [6] published an exponential shear deformation theory for the free vibration of thick isotropic plates with exponential terms in the displacement field to calculate the stresses and the strain. This theory is compatible with stress free boundary conditions at the top and the bottom of the plate. Sayyad [7] proposed a refined shear deformation theory for the static flexure and free vibration analysis of thick isotropic beams, considering sinusoidal, hyperbolic and exponential functions in terms of thickness co-ordinate associated with transverse shear deformation effect. There is also a consistent higher-order shear deformation non-linear theory for shells of generic shape by Amabili and Reddy [8], which also considers geometric imperfections. Completely non-linear terms for the in-plane displacements were used to calculate the geometrically non-linear strain–displacement relationships. Another consistent higher-order shear deformation nonlinear theory for shells of generic shape was developed by Amabili [9]. It is also compatible with geometric imperfections and permits the variation of thickness by using six variables. This new theory was used for laminated circular cylindrical shells complete around the circumference and simply supported at the ends. Daouadji et al. [10] presented Navier’s solutions of rectangular plates originating from a new higher order shear deformation model for the static response of functionally graded plates, enforcing traction-free boundary conditions at plate surfaces. A correct representation of transverse shearing strain made shear correction factors unnecessary. Thai and Vo [11] proposed a new sinusoidal shear deformation theory for bending, buckling, and vibration of functionally graded plates. The theory accounted for sinusoidal distribution of transverse shear stress. Unlike the conventional sinusoidal shear deformation theory, the suggested sinusoidal shear deformation theory contains only four unknowns and is very similar to classical plate theory expressions. El Meiche et al. [12] investigated a new hyperbolic shear deformation theory, which also takes into consideration transverse shear deformation effects for the buckling and free vibration analysis of thick functionally graded sandwich plates. Unlike any other theory, this theory needs only four governing equations. The authors assumed that the variation of the plate properties was a consequence of plate thickness following a simple power law distribution in terms of volume fraction of material constituents. In this theory, in order to satisfy the boundary conditions on free surfaces, the authors assumed that the shear stresses change with parabolic distribution.

To the best of authors’ knowledge, most of the above theories have not been used for studying nanostructures yet. So it may be useful to develop a new model based on one of these new theories for rectangular nanoplates. In this work, the bending analysis of nanoplates based on nonlocal elasticity theory using the trigonometric shear deformation theory is investigated. After validating the formulation and the method of solution, the small scale effect with the influences of geometrical shape on the static behavior of the rectangular nanoplates is also investigated.

## Review of nonlocal elasticity theory

Based on the nonlocal elasticity theory, the points can have translational motion similar to the classical theories, but the stress field at an arbitrary point in an elastic medium depends on the strain field in a place near that point [13]. The constitutive relations on the basis of nonlocal elasticity theory for a graphene sheet represented by the following differential equations [14–15]:

[1]
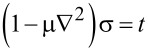


Where μ is the nonlocal parameter, *t* is the macroscopic stress tensor at a point, σ is the nonlocal stress tensor and


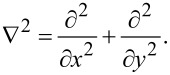


Above equations can be written as follow,

[2]
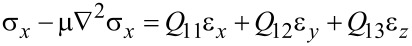


[3]
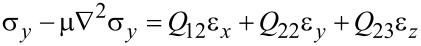


[4]
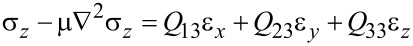


[5]
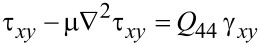


[6]
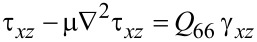


[7]
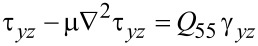


Where *Q*_ij_ are the elastic constant and σ_i_ and τ_ij_ are the normal and shear stresses, respectively.

## Governing equations

We use rectangular cartesian coordinates to describe deformations of isotropic nanoplates. Our governing equations are derived based on trigonometric shear deformation theory in conjunction with nonlocal elasticity theory. The calculation is available in detail in the Supporting Information File 1. The difference between the nondimensional deflections and deflection ratios will be discussed in the next section.

## Numerical results

First of all, to show the reliability and accuracy of the aforementioned equations, our numerical results are compared with those available in literature for simply supported macro plates. In Table 1, the present results are compared with several other works for different length to thickness ratios. Different methodologies [5,16–20] such as exact solution [16] and higher order shear deformation theory [18] are also listed in Table 1. One can see that our results are in a good agreement with exact solutions.

**Table 1:** Transverse deflections for square macro plate under uniform load.

Method of solution									
a/h	exact [16]	present method	Mantari et al. [5]	Reddy [17]	Ferreira et al. [18]	Ferreira et al. [19]	Karama [20]	Touratier [20]	Reddy [20]

10	4.79	4.78	4.67	4.77	4.79	4.79	4.61	4.61	4.61
20	4.57	4.59	4.59	4.57	4.61	4.62	4.43	4.44	4.44
50	4.50	4.54	4.45	4.50	4.58	4.58	4.39	4.40	4.35

**Figure 1:**
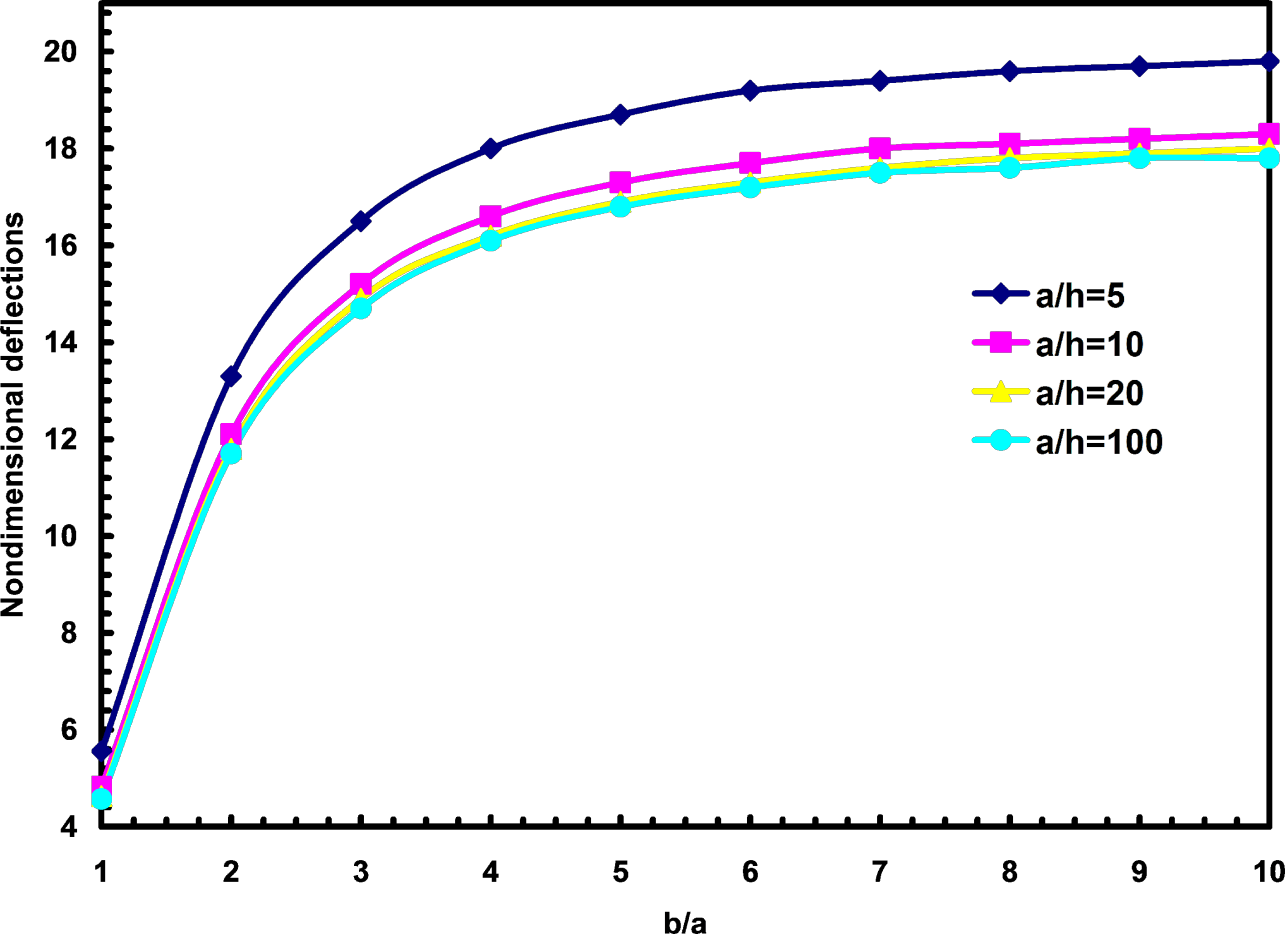
The effects of aspect ratio and length to thickness ratio on the nondimensional deflections.

The second observation is the effects of the aspect ratio and length to thickness ratio on the nondimensional deflections, as shown in Figure 1. It is observed that with the increase of length to thickness ratio, the deflections will decrease. It is also shown that increasing the aspect ratio will increase the deflection. It seems that for b/a > 10, the aspect ratio doesn’t play an important role in our discussion. From this figure, one can find that the geometrical shape in studying nanoplates is as much important as in investigating macro plates. It should be mentioned that all the nanoplates in this section are subjected to uniformly distributed loading unless otherwise specified.

**Figure 2:**
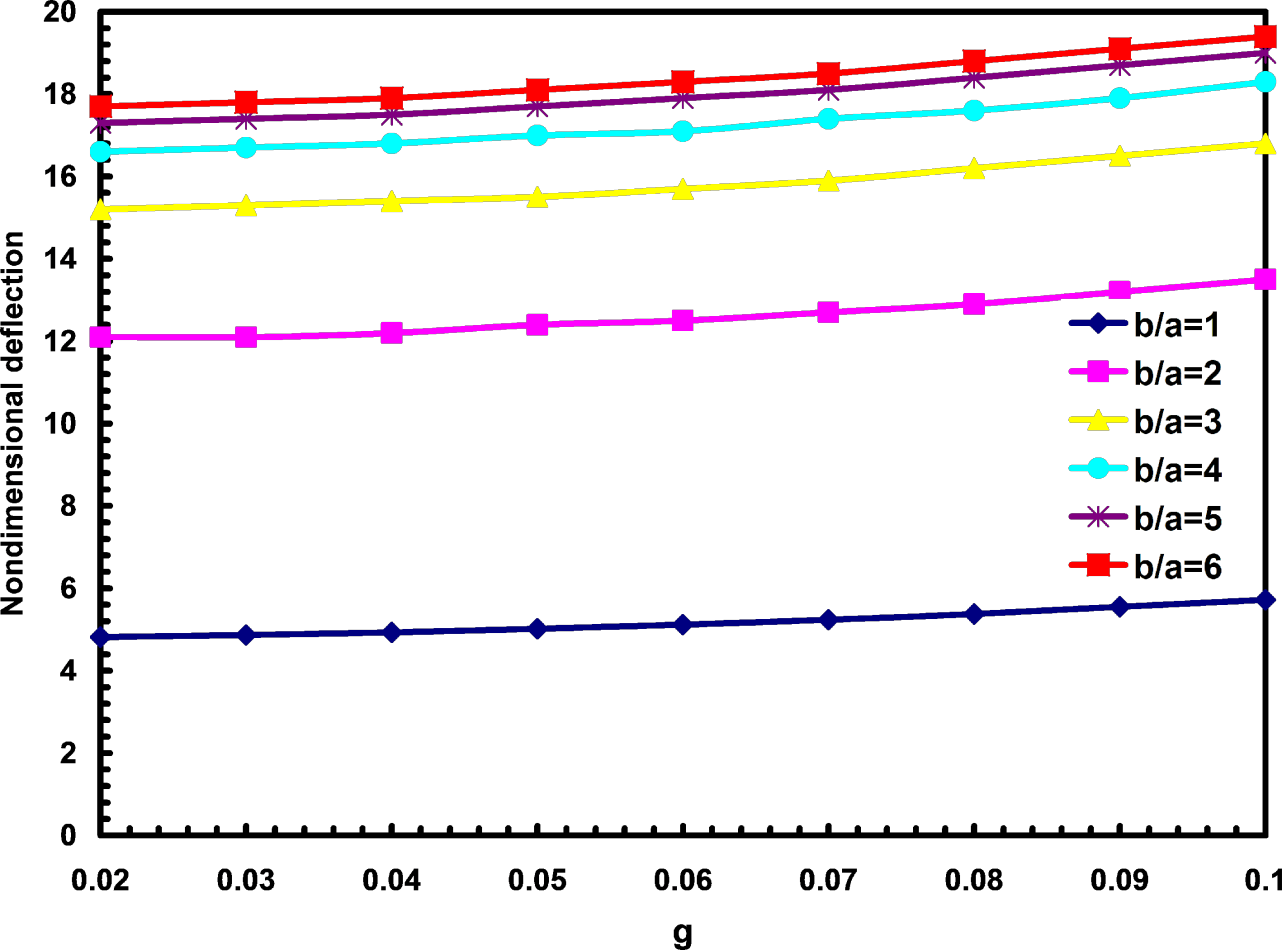
The effects of nonlocal parameter and aspect ratio on the nondimensional deflections.

Figure 2 depicts the effects of nonlocal parameter on the nondimensional deflections for different aspect ratios. In this figure the nondimensional parameter g is defined 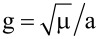. It is found that with the increase of nonlocal parameter, the nondimensional deflections are increased for all aspect ratios. From this figure, one can also find that the aspect ratio has more effects in comparison with parameter g. It is also figured that the deflections of square nanoplates are less than those for rectangular nanoplates for different nonlocal parameters.

**Figure 3:**
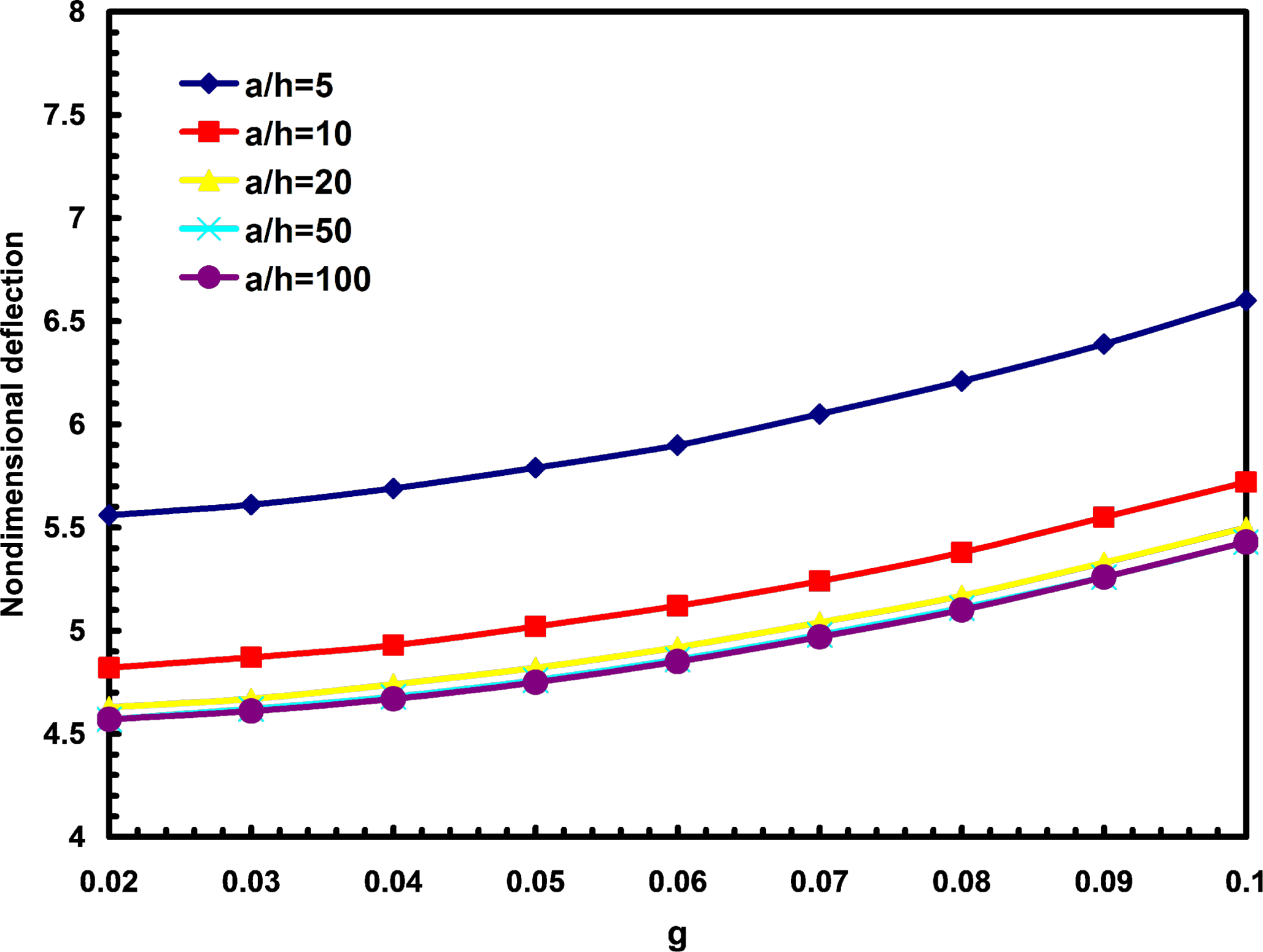
The effects of nonlocal parameter and length to thickness ratio on the nondimensional deflection.

As another example, the influences of parameter g and length to thickness ratio are considered in Figure 3. It is found that for thin, moderately thick and thick rectangular nanoplates, increasing the nonlocal parameter will cause increasing the nondimensional deflections. It is also obtained that the influences of nonlocal parameter cannot be neglected so the classical plate theories for macro plates are not suitable for studying nanoplates.

To compare the results of macro and nanoplates and studying the size effects, one new parameter was defined by researchers as,


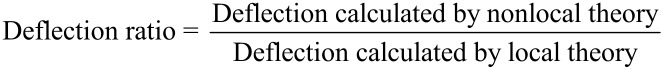


**Figure 4:**
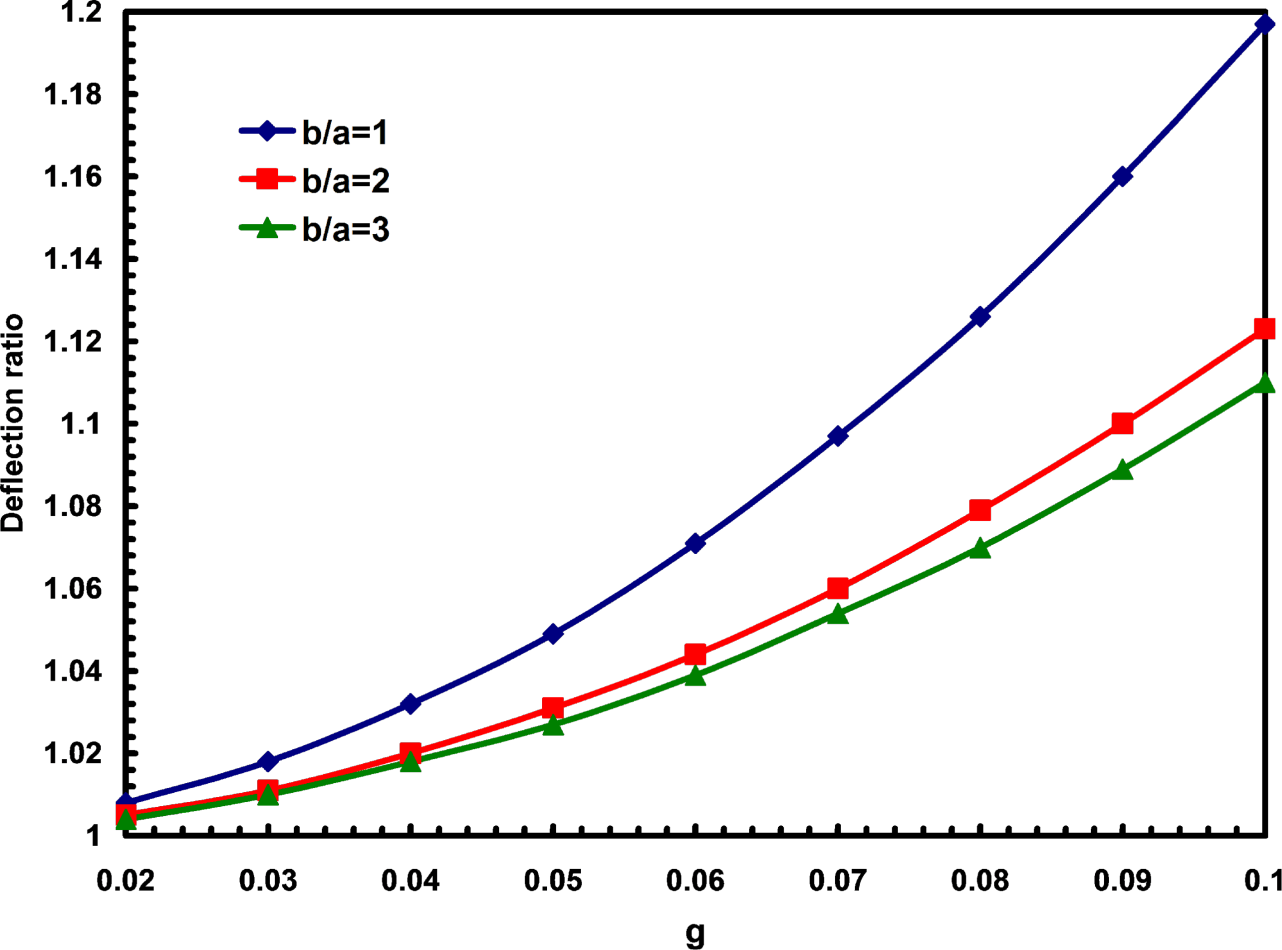
The effects of nonlocal parameter and aspect ratio on the deflection ratios.

It is obvious that the deflection ratio for macro plates is 1. In Figure 4, the influences of both aspect ratio and parameter g on the deflection ratios are presented. It is observed that with the increase of nonlocal parameter, the deflection ratios increase. It is also shown that for higher aspect ratios, the effects of nonlocal parameter on deflection ratios will decrease.

**Figure 5:**
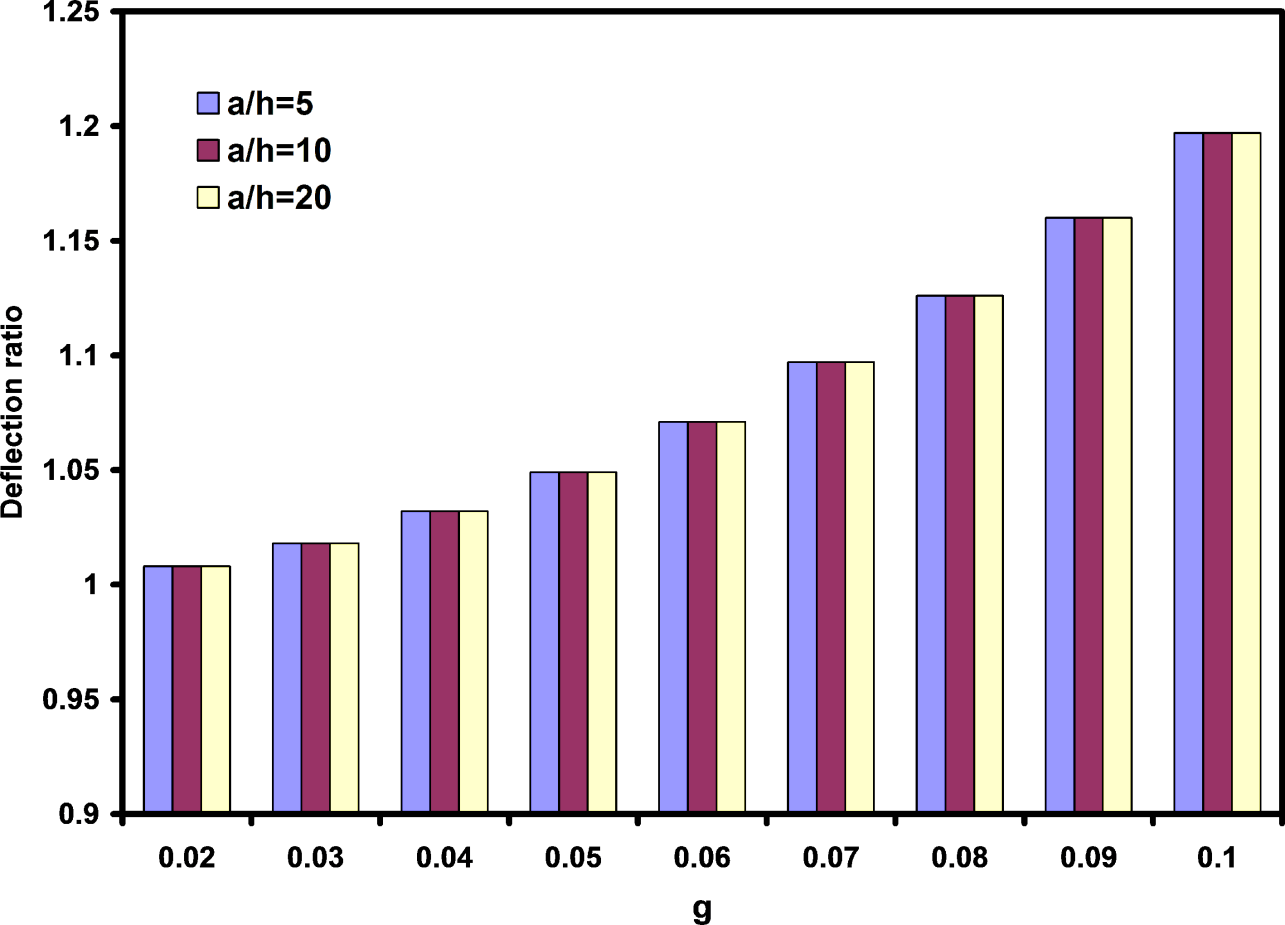
The effects of nonlocal parameter and length to thickness ratio on the deflection ratios.

One of the major differences between nondimensional deflections and deflection ratios is illustrated in Figure 5. In this figure, the effects of length to thickness ratio and parameter g are investigated. It is obtained that the length to thickness ratio has no significant effect on the deflection ratios. Similar results were seen by other researchers for frequency ratio [21].

**Figure 6:**
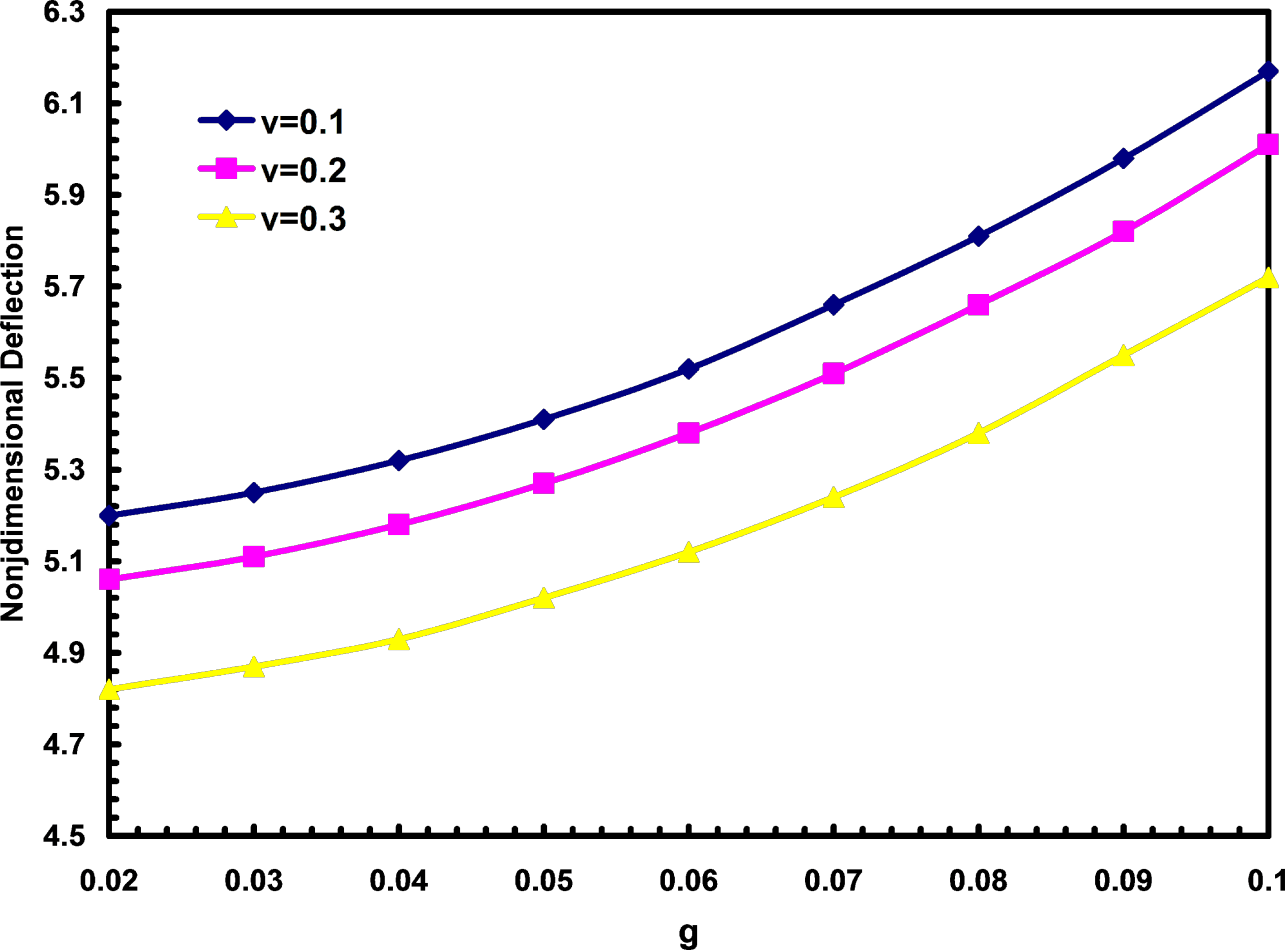
The effects of nonlocal parameter and Poisson's ratio on the nondimensional deflections.

**Figure 7:**
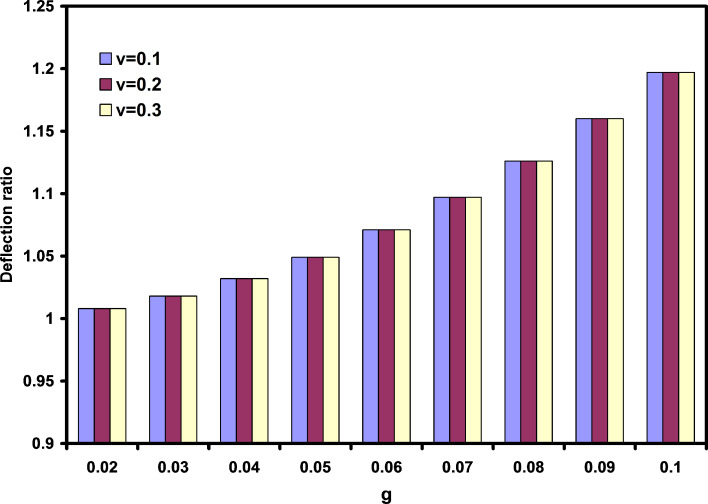
The effects of nonlocal parameter and Poisson's ratio on the deflection ratios.

Another difference between deflection ratios and nondimensional deflections are shown in Figure 6 and Figure 7. In these figures the influences of Poisson's ratio are studied. It is shown that increasing the Poisson's ratio will decrease the nondimensional deflections but with the increase of Poisson's ratio, the deflection ratio will remain constant. As it is obtained in Figure 6 and Figure 7, above result is not affected by changing the nonlocal parameter.

**Figure 8:**
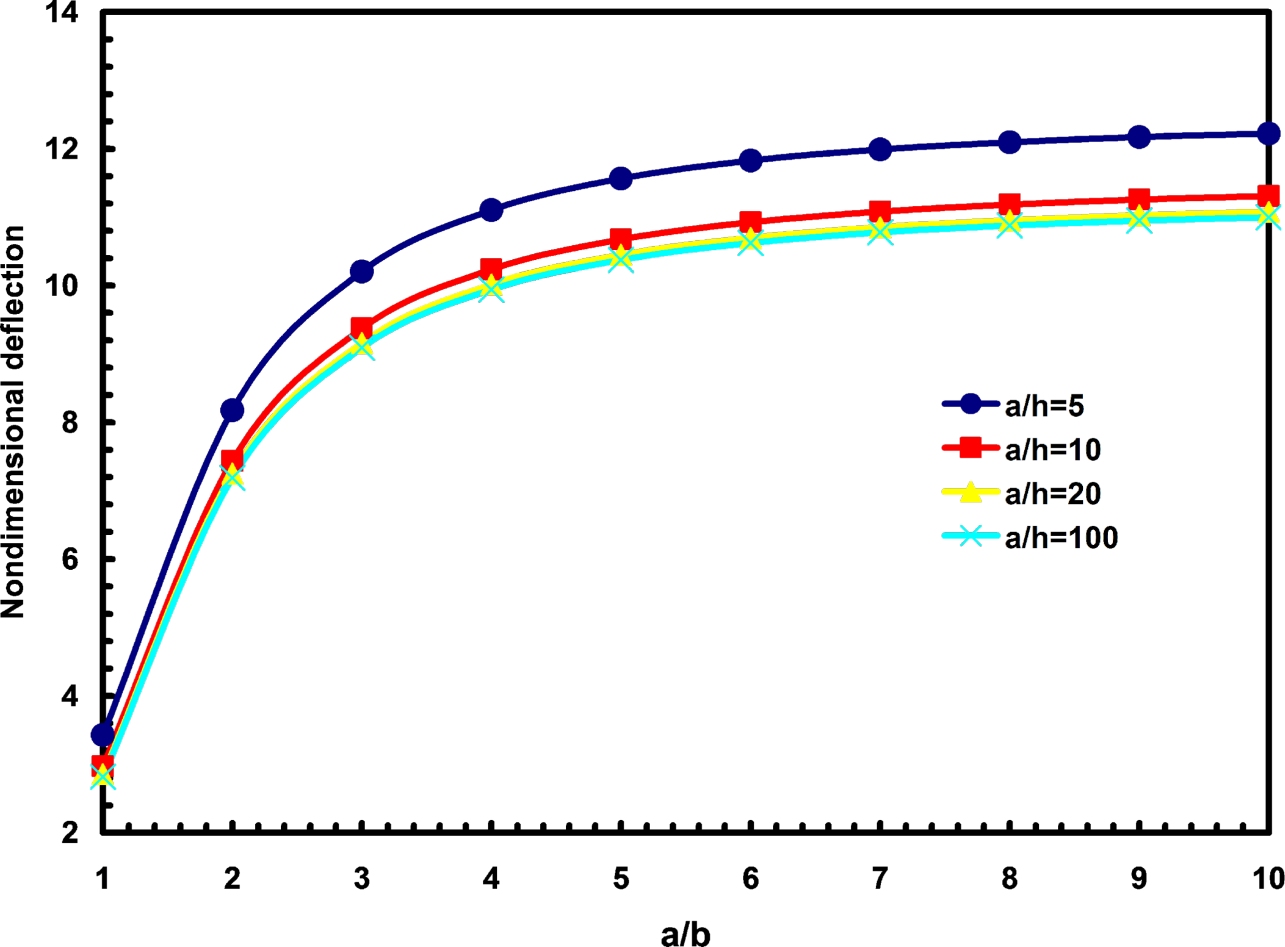
The effects of aspect ratio and length to thickness ratio on the nondimensional deflections under sinusoidal loading.

Figure 8 depicts the influences of both aspect ratio and length to thickness ratio on the nondimensional deflections under sinusoidal loading. It can be seen that with the increase of length to thickness ratio, the deflections will decrease. It is also shown that increasing the aspect ratio will increase the deflections. It is mentioned that the nondimensional deflections for nanoplate under sinusoidal loading are less than those for nanoplates subjected to uniform load, as shown in Figure 1 and Figure 8.

## Conclusion

In this paper, the bending analysis of simply supported rectangular nanoplates was analytically investigated. Our formulations were based on trigonometric shear deformation theory and nonlocal elasticity theory. The effects of different parameters such as nonlocal parameter and geometrical shape on the nondimensional deflections and deflection ratios were studied. It was shown that the behavior of deflection ratios and nondimensional deflections are not the same and they should investigate separately. It was also obtained that the effects of length to thickness ratio and Poisson's ratio can be ignored in studying deflection ratios.

## Supporting Information

File 1 Governing equations.
