# Supplementary material for: Static analysis of rectangular nanoplates using trigonometric shear deformation theory based on nonlocal elasticity theory
Source: Beilstein J Nanotechnol. 2013 Dec 30;4:968–73. doi: 10.3762/bjnano.4.109 (PMC3896292; doi:10.3762/bjnano.4.109)
Supplement: File 1 — Governing equations. [file Beilstein_J_Nanotechnol-04-968-s001.pdf]

Supporting Information

for

Static analysis of rectangular nanoplates using trigonometric shear deformation theory based on nonlocal elasticity theory

Mohammad Rahim Nami and Maziar Janghorban*

Address: School of Mechanical Engineering, Shiraz University, Shiraz, Iran

Email: Maziar Janghorban - maziar.janghorban@gmail.com

* Corresponding author

Governing equations

In this section, we use rectangular cartesian coordinates to describe deformations of isotropic nanoplates. Our governing equations are derived based on trigonometric shear deformation theory in conjunction with nonlocal elasticity theory. We begin with the displacement field [1],

$$
\begin{aligned}
u(x,y,z,t) &= -z\frac{\partial w(x,y,t)}{\partial x}+\frac{h}{\pi}\sin\frac{\pi z}{h}Q(x,y,t)\\
v(x,y,z,t) &= -z\frac{\partial w(x,y,t)}{\partial y}+\frac{h}{\pi}\sin\frac{\pi z}{h}\psi(x,y,t)\\
w(x,y,z,t) &= w(x,y,t)+\frac{h}{\pi}\cos\frac{\pi z}{h}\xi(x,y,t)
\end{aligned}
\tag{8}
$$

One can easily find that the present theory is based on four unknown parameter (w, Q, ψ, ξ). The linear strain-displacement relations for trigonometric theory are defined as follow [1],

$$
\begin{aligned}
\varepsilon_x &= \frac{\partial u}{\partial x} = -z\frac{\partial^2 w}{\partial x^2} + \frac{h}{\pi}\sin\frac{\pi z}{h}\frac{\partial Q}{\partial x} \\
\varepsilon_y &= \frac{\partial v}{\partial y} = -z\frac{\partial^2 w}{\partial y^2} + \frac{h}{\pi}\sin\frac{\pi z}{h}\frac{\partial \psi}{\partial y} \\
\varepsilon_z &= \frac{\partial w}{\partial z} = -\xi\sin\frac{\pi z}{h} \\
\gamma_{xy} &= \frac{\partial u}{\partial y} + \frac{\partial v}{\partial x} = -2z\frac{\partial^2 w}{\partial x\partial y} + \frac{h}{\pi}\sin\frac{\pi z}{h}\left(\frac{\partial Q}{\partial y} + \frac{\partial \psi}{\partial x}\right) \\
\gamma_{xz} &= \frac{\partial u}{\partial z} + \frac{\partial w}{\partial x} = \cos\frac{\pi z}{h}\left(\frac{h}{\pi}\frac{\partial \xi}{\partial x} + Q\right) \\
\gamma_{yz} &= \frac{\partial v}{\partial z} + \frac{\partial w}{\partial y} = \cos\frac{\pi z}{h}\left(\frac{h}{\pi}\frac{\partial \xi}{\partial y} + \psi\right)
\end{aligned}
\tag{9}
$$

According to the principle of virtual displacements to derive the equilibrium equations appropriate for the displacement field in Equation 8 and constitutive equations in Equation 1, the following equilibrium equations can be obtained [1],

$$
\begin{aligned}
&\frac{\partial^2 M_x}{\partial x^2} + 2\frac{\partial^2 M_{xy}}{\partial x\partial y} + \frac{\partial^2 M_y}{\partial y^2} + q = 0 \\
&\frac{\partial M_{sx}}{\partial x} + \frac{\partial V_{sxy}}{\partial y} - \frac{\pi}{h}V_{sx} = 0 \\
&\frac{\partial M_{sy}}{\partial y} + \frac{\partial V_{sxy}}{\partial x} - \frac{\pi}{h}V_{sy} = 0 \\
&\frac{\partial V_{sx}}{\partial x} + \frac{\partial V_{sy}}{\partial y} - \frac{\pi}{h}V_{sz} = 0
\end{aligned}
\tag{10}
$$

where,

$$
\begin{bmatrix} M_x \\ M_y \\ M_{xy} \end{bmatrix} = \int_{-h/2}^{h/2} \begin{Bmatrix} \sigma_x \\ \sigma_y \\ \tau_{xy} \end{Bmatrix} z\,dz \qquad
\begin{bmatrix} M_{sx} \\ M_{sy} \\ V_{sxy} \\ V_{sz} \end{bmatrix} = \int_{-h/2}^{h/2} \begin{Bmatrix} \sigma_x \\ \sigma_y \\ \tau_{xy} \\ \sigma_z \end{Bmatrix} \frac{h}{\pi}\sin\frac{\pi z}{h}\,dz
$$

$$
\begin{bmatrix} V_{sx} \\ V_{sy} \end{bmatrix} = \int_{-h/2}^{h/2} \begin{Bmatrix} \tau_{zx} \\ \tau_{zy} \end{Bmatrix} \left(\frac{h}{\pi}\cos\frac{\pi z}{h}\right) dz
$$

(11)

In order to account the size effects in conjunction with equilibrium Equations 10, following integrations are needed

$\int_{-h/2}^{h/2} (equation(2))\, zdz:$

$$M_x - \mu\nabla^2 M_x = Q_{11}\left(-F_3\frac{\partial^2 w}{\partial x^2} + A_0\frac{\partial Q}{\partial x}\right) + Q_{12}\left(-F_3\frac{\partial^2 w}{\partial y^2} + A_0\frac{\partial \psi}{\partial y}\right) + Q_{13}\left(-\xi\frac{\pi}{h}A_0\right)$$

(12)

$\int_{-h/2}^{h/2} (equation(3))\, zdz:$

$$M_y - \mu\nabla^2 M_y = Q_{12}\left(-F_3\frac{\partial^2 w}{\partial x^2} + A_0\frac{\partial Q}{\partial x}\right) + Q_{22}\left(-F_3\frac{\partial^2 w}{\partial y^2} + A_0\frac{\partial \psi}{\partial y}\right) + Q_{23}\left(-\xi\frac{\pi}{h}A_0\right)$$

(13)

$\int_{-h/2}^{h/2} (equation(5))\, zdz:$

$$M_{xy} - \mu\nabla^2 M_{xy} = Q_{44}\left(-2F_3\frac{\partial^2 w}{\partial x\partial y} + A_0\left(\frac{\partial Q}{\partial y} + \frac{\partial \psi}{\partial x}\right)\right)$$

(14)

$\int_{-h/2}^{h/2} (equation(2))\frac{h}{\pi}\sin\frac{\pi z}{h} dz:$

$$M_{sx} - \mu\nabla^2 M_{sx} = Q_{11}\left(-A_0\frac{\partial^2 w}{\partial x^2} + B_0\frac{\partial Q}{\partial x}\right) + Q_{12}\left(-A_0\frac{\partial^2 w}{\partial y^2} + B_0\frac{\partial \psi}{\partial y}\right) + Q_{13}\left(-\xi\frac{\pi}{h}B_0\right)$$

(15)

$\int_{-h/2}^{h/2} (equation(3))\frac{h}{\pi}\sin\frac{\pi z}{h} dz:$

$$M_{sy} - \mu\nabla^2 M_{sy} = Q_{12}\left(-A_0\frac{\partial^2 w}{\partial x^2} + B_0\frac{\partial Q}{\partial x}\right) + Q_{22}\left(-A_0\frac{\partial^2 w}{\partial y^2} + B_0\frac{\partial \psi}{\partial y}\right) + Q_{23}\left(-\xi\frac{\pi}{h}B_0\right)$$

(16)

$\int_{-h/2}^{h/2} (equation(5))\frac{h}{\pi}\sin\frac{\pi z}{h} dz:$

$$V_{sxy} - \mu\nabla^2 V_{sxy} = Q_{44}\left(-2A_0\frac{\partial^2 w}{\partial x\partial y} + B_0\left(\frac{\partial Q}{\partial y} + \frac{\partial \psi}{\partial x}\right)\right)$$

(17)

$$\int_{-h/2}^{h/2} (equation(4))\frac{h}{\pi}\sin\frac{\pi z}{h}dz:$$

$$V_{sz} - \mu\nabla^2 V_{sz} = Q_{13}\left(-A_0\frac{\partial^2 w}{\partial x^2} + B_0\frac{\partial Q}{\partial x}\right) + Q_{23}\left(-A_0\frac{\partial^2 w}{\partial y^2} + B_0\frac{\partial \psi}{\partial y}\right) + Q_{33}\left(-\xi\frac{\pi}{h}B_0\right) \quad (18)$$

$$\int_{-h/2}^{h/2} \left(equation(10)\right)\left(\frac{h}{\pi}\cos\frac{\pi z}{h}\right)dz:$$

$$V_{sx} - \mu\nabla^2 V_{sx} = Q_{66}\left(C_0\frac{\partial \xi}{\partial x} + \frac{\pi}{h}C_0 Q\right) \quad (19)$$

$$\int_{-h/2}^{h/2} \left(equation(11)\right)\left(\frac{h}{\pi}\cos\frac{\pi z}{h}\right)dz:$$

$$V_{sy} - \mu\nabla^2 V_{sy} = Q_{55}\left(C_0\frac{\partial \xi}{\partial y} + C_0\frac{\pi}{h}\psi\right) \quad (20)$$

where $F_3 = \int_{-h/2}^{h/2} z^2 dz$ $\quad A_0 = \left[\int_{-h/2}^{h/2} z\sin\frac{\pi z}{h}dz\right]\frac{h}{\pi}$ $\quad B_0 = \int_{-h/2}^{h/2}\left(\frac{h}{\pi}\right)^2 \sin^2\frac{\pi z}{h}dz$. By differentiating from above Equations 12–17 and Equations 19 and 20, the format of governing Equations 10 can be achieved easily.

$$\frac{\partial^2}{\partial x^2}\left(equation(12)\right):$$

$$\frac{\partial^2 M_x}{\partial x^2} - \mu\nabla^2\left(\frac{\partial^2 M_x}{\partial x^2}\right) = Q_{11}\left(-F_3\frac{\partial^4 w}{\partial x^4} + A_0\frac{\partial^3 Q}{\partial x^3}\right) + Q_{12}\left(-F_3\frac{\partial^4 w}{\partial x^2\partial y^2} + A_0\frac{\partial^3 \psi}{\partial y\partial x^2}\right) - Q_{13}A_0\left(\frac{\pi}{h}\right)\frac{\partial^2 \xi}{\partial x^2} \quad (21)$$

$$\frac{\partial^2}{\partial y^2}\left(equation(13)\right):$$

$$\frac{\partial^2 M_y}{\partial y^2} - \mu\nabla^2\left(\frac{\partial^2 M_y}{\partial y^2}\right) = Q_{12}\left(-F_3\frac{\partial^4 w}{\partial x^2\partial y^2} + A_0\frac{\partial^3 Q}{\partial x\partial y^2}\right) + Q_{22}\left(-F_3\frac{\partial^4 w}{\partial x^4} + A_0\frac{\partial^3 \psi}{\partial y^3}\right) - \left(\frac{\pi}{h}\right)Q_{23}A_0\frac{\partial^2 \xi}{\partial y^2} \quad (22)$$

$$2\frac{\partial^2}{\partial x\partial y}\left(equation(14)\right):$$

$$2\frac{\partial^2 M_{xy}}{\partial x\partial y}-\mu\nabla^2\left(2\frac{\partial^2 M_{xy}}{\partial x\partial y}\right)=2Q_{44}\left(-2F_3\frac{\partial^4 w}{\partial x^2\partial y^2}+A_0\left(\frac{\partial^3 Q}{\partial x\partial y^2}+\frac{\partial^3\psi}{\partial y\partial x^2}\right)\right)$$

(23)

$\frac{\partial}{\partial x}\left(equation(15)\right):$

$$\frac{\partial^2 M_{sx}}{\partial x}-\mu\nabla^2\left(\frac{\partial^2 M_{sx}}{\partial x}\right)=Q_{11}\left(-A_0\frac{\partial^3 w}{\partial x^3}+B_0\frac{\partial^2 Q}{\partial x^2}\right)+Q_{12}\left(-A_3\frac{\partial^3 w}{\partial x\partial y^2}+B_0\frac{\partial^2\psi}{\partial x\partial y}\right)-Q_{13}B_0\left(\frac{\pi}{h}\right)\frac{\partial\xi}{\partial x}$$

(24)

$\frac{\partial}{\partial y}\left(equation(17)\right):$

$$2\frac{\partial^2 V_{sxy}}{\partial y}-\mu\nabla^2\left(\frac{\partial V_{sxy}}{\partial y}\right)=Q_{44}\left(-2A_0\frac{\partial^3 w}{\partial x\partial y^2}+B_0\left(\frac{\partial^2 Q}{\partial y^2}+\frac{\partial^2\psi}{\partial y\partial x}\right)\right)$$

(25)

$\frac{\partial}{\partial x}\left(equation(17)\right):$

$$\frac{\partial V_{sxy}}{\partial x}-\mu\nabla^2\left(\frac{\partial V_{sxy}}{\partial x}\right)=Q_{44}\left(-2A_0\frac{\partial^3 w}{\partial x\partial y^2}+B_0\left(\frac{\partial^2 Q}{\partial x\partial y}+\frac{\partial^2\psi}{\partial x^2}\right)\right)$$

(26)

$\frac{\partial}{\partial x}\left(equation(19)\right):$

$$\frac{\partial V_{sx}}{\partial x}-\mu\nabla^2\left(\frac{\partial V_{sx}}{\partial x}\right)=Q_{66}\left(C_0\frac{\partial^2\xi}{\partial x^2}+\frac{\pi}{h}C_0\frac{\partial Q}{\partial x}\right)$$

(27)

$\frac{\partial}{\partial y}\left(equation(20)\right):$

$$\frac{\partial V_{sy}}{\partial y}-\mu\nabla^2\left(\frac{\partial V_{sy}}{\partial y}\right)=Q_{55}\left(C_0\frac{\partial^2\xi}{\partial y^2}+C_0\frac{\pi}{h}\frac{\partial\psi}{\partial y}\right)$$

(28)

$\frac{\partial}{\partial y}\left(equation(16)\right):$

$$\frac{\partial Msy}{\partial y}-\mu\nabla^2\frac{\partial Msy}{\partial y}=Q_{12}\left(-A_0\frac{\partial^3 w}{\partial y\partial x^2}+B_0\frac{\partial^2 Q}{\partial x\partial y}\right)+Q_{22}\left(-A_0\frac{\partial^3 w}{\partial y^3}+B_0\frac{\partial^2\psi}{\partial y^2}\right)-\left(\frac{\pi}{h}\right)Q_{23}B_0\frac{\partial\xi}{\partial y}$$

(29)

Where $C_0=\int_{-h/2}^{h/2}\left(\frac{h}{\pi}\right)^2\cos^2\frac{\pi z}{h}dz$. By using the classical equilibrium Equations 10 and the above equations with considering size effects, four governing equations for investigating the static analysis of rectangular nanoplates can be expressed in terms of the unknown displacement components as follow,

$(21)+(23)+(22)$:

$$Q_{11}\left(-F_3\frac{\partial^4 w}{\partial x^4}+A_0\frac{\partial^3 Q}{\partial x^3}\right)+Q_{12}\left(-F_3\frac{\partial^4 w}{\partial x^2\partial y^2}+A_0\frac{\partial^3\psi}{\partial y\partial x^2}\right)+Q_{12}\left(-F_3\frac{\partial^4 w}{\partial x^2\partial y^2}+A_0\frac{\partial^3 Q}{\partial x\partial y^2}\right)$$
$$+Q_{22}\left(-F_3\frac{\partial^4 w}{\partial y^4}+A_0\frac{\partial^3\psi}{\partial y^3}\right)+2Q_{44}\left(-2F_3\frac{\partial^4 w}{\partial x^2\partial y^2}+A_0\left(\frac{\partial^3 Q}{\partial x\partial y^2}+\frac{\partial^3\psi}{\partial y\partial x^2}\right)\right)-\frac{\pi}{h}Q_{13}A_0\frac{\partial^2\xi}{\partial x^2}$$
$$-\frac{\pi}{h}Q_{23}A_0\frac{\partial^2\xi}{\partial y^2}=(-q)-\mu\nabla^2(-q)$$

(30)

$(24)+(25)-\frac{\pi}{h}(19)$:

$$Q_{11}\left(-A_0\frac{\partial^3 w}{\partial x^3}+B_0\frac{\partial^2 Q}{\partial x^2}\right)+Q_{12}\left(-A_0\frac{\partial^3 w}{\partial x\partial y^2}+B_0\frac{\partial^2\psi}{\partial x\partial y}\right)$$
$$+Q_{44}\left(-2A_0\frac{\partial^3 w}{\partial x\partial y^2}+B_0\left(\frac{\partial^2 Q}{\partial y^2}+\frac{\partial^2\psi}{\partial x\partial y}\right)\right)-\frac{\pi}{h}Q_{66}\left(C_0\frac{\partial\xi}{\partial x}+\frac{\pi}{h}C_0 Q\right)-\frac{\pi}{h}Q_{13}B_0\frac{\partial\xi}{\partial x}=0$$

(31)

$(29)+(26)-\frac{\pi}{h}(20)$:

$$Q_{12}\left(-A_0\frac{\partial^3 w}{\partial y\partial x^2}+B_0\frac{\partial^2 Q}{\partial x\partial y}\right)+Q_{22}\left(-A_0\frac{\partial^3 w}{\partial y^3}+B_0\frac{\partial^2\psi}{\partial y^2}\right)+Q_{44}\left(-2A_0\frac{\partial^3 w}{\partial y\partial x^2}+B_0\left(\frac{\partial^2 Q}{\partial x\partial y}+\frac{\partial^2\psi}{\partial x^2}\right)\right)$$
$$-\frac{\pi}{h}Q_{55}C_0\left(\frac{\partial\xi}{\partial y}+\frac{\pi}{h}\psi\right)-\frac{\pi}{h}Q_{23}B_0\frac{\partial\xi}{\partial y}=0$$

(32)

$$(27)+(28)-\frac{\pi}{h}(18):$$

$$Q_{66}\left(C_0\frac{\partial^2\xi}{\partial x^2}+\frac{\pi}{h}C_0\frac{\partial Q}{\partial x}\right)+Q_{55}\left(C_0\frac{\partial^2\xi}{\partial y^2}+C_0\frac{\pi}{h}\frac{\partial\psi}{\partial y}\right)$$
$$-\frac{\pi}{h}\left(Q_{13}\left(-A_0\frac{\partial^2 w}{\partial x^2}+B_0\frac{\partial Q}{\partial x}\right)+Q_{23}\left(-A_0\frac{\partial^2 w}{\partial y^2}+B_0\frac{\partial\psi}{\partial y}\right)+Q_{33}\left(-\xi\frac{\pi}{h}B_0\right)\right)=0$$

(33)

It can be seen from the above equation that the classical or local plate theory is recovered when the parameter μ is set identically to zero. It may be important to mention that the above formulations are general and can be used for symmetric anisotropic nanoplates. According to our knowledge about analytical methods for macro plates, we can employ the following displacements for simply supported rectangular nanoplates as,

$$W=\sum_{m=1}^{\infty}\sum_{n=1}^{\infty}W_{mn}\sin\alpha x\sin\beta y$$
$$Q=\sum_{m=1}^{\infty}\sum_{n=1}^{\infty}Q_{mn}\cos\alpha x\sin\beta y$$
$$\psi=\sum_{m=1}^{\infty}\sum_{n=1}^{\infty}\psi_{mn}\sin\alpha x\cos\beta y$$
$$\xi=\sum_{m=1}^{\infty}\sum_{n=1}^{\infty}\xi_{mn}\sin\alpha x\sin\beta y$$

(34)

These displacements are automatically satisfied the simply supported boundary conditions. By using the above displacement field in governing equations, one can obtain the following equations,

$$(30):$$
$$\left(-Q_{11}F_3\alpha^4-2Q_{12}F_3\alpha^2\beta^2-Q_{22}F_3\beta^4-4F_3Q_{44}\alpha^2\beta^2\right)W_{mn}+\left(Q_{11}A_0\alpha^3+Q_{12}A_0\alpha\beta^2+2Q_{44}A_0\alpha\beta^2\right)Q_{mn}$$
$$+\left(Q_{12}A_0\beta\alpha^2+Q_{22}A_0\beta^3+2Q_{44}A_0\beta\alpha^2\right)\psi_{mn}+\frac{\pi}{h}(Q_{13}A_0\alpha^2+Q_{23}A_0\beta^2)\xi_{mn}=-q-\mu(\alpha^2+\beta^2)q$$

(35)

(31):

$$\left(Q_{11}A_0\alpha^3+Q_{12}A_0\alpha\beta^2+2Q_{44}A_0\alpha\beta^2\right)W_{mn}+\left(-Q_{11}B_0\alpha^2-Q_{44}B_0\beta^2-(\frac{\pi}{h})^2Q_{66}C_0\right)Q_{mn}$$
$$+\left(-Q_{12}B_0\alpha\beta-Q_{44}B_0\alpha\beta\right)\psi_{mn}+\left(-\frac{\pi}{h}Q_{66}C_0\alpha-\frac{\pi}{h}Q_{13}B_0\alpha\right)\xi_{mn}=0 \tag{36}$$

(32):

$$\left(Q_{12}A_0\alpha^2\beta+Q_{22}A_0\beta^3+2Q_{44}A_0\alpha^2\beta\right)W_{mn}+\left(-Q_{12}B_0\alpha\beta-Q_{44}B_0\alpha\beta\right)Q_{mn}$$
$$+\left(-Q_{22}B_0\beta^2-Q_{44}B_0\alpha^2-\frac{\pi^2}{h^2}Q_{55}C_0\right)\psi_{mn}+\left(-\frac{\pi}{h}Q_{55}C_0\beta-\frac{\pi}{h}Q_{23}B_0\beta\right)\xi_{mn}=0 \tag{37}$$

(33):

$$\left(-\frac{\pi}{h}Q_{13}A_0\alpha^2-\frac{\pi}{h}Q_{23}A_0\beta^2\right)W_{mn}+\left(-\frac{\pi}{h}Q_{66}C_0\alpha+\frac{\pi}{h}Q_{13}B_0\alpha\right)Q_{mn}$$
$$+\left(-C_0Q_{55}\frac{\pi}{h}\beta+\frac{\pi}{h}Q_{23}B_0\beta\right)\psi_{mn}+\left(-Q_{66}C_0\alpha^2-Q_{55}C_0\beta^2+Q_{33}(\frac{\pi}{h})^2B_0\right)\xi_{mn}=0 \tag{38}$$

Now by using MATLAB software, the nondimensional deflections and deflection ratios of rectangular nanoplates can be obtained. The difference between the nondimensional deflections and deflection ratios will be discussed in the next section of the main manuscript.

References

1. Ghugal, Y. M.; Sayyad, A. S. *LAJSS* **2011,** *8,* 229–243.
